# Bioactive and antioxidant properties in highbush blueberry cultivars: identifying superior cultivars for nutritional biofortification

**DOI:** 10.3389/fpls.2025.1750179

**Published:** 2026-01-12

**Authors:** Azam Akbari, Yige Xu, Xin Wei, Zichen Liu, Doğan Ergün, Cihan Akgöl, Feiyu Dong, Sezai Ercişli, Salih Kafkas, Cheng Liu, Nesibe Ebru Kafkas

**Affiliations:** 1 Department of Horticulture, Faculty of Agriculture, Cukurova University, Adana, Türkiye; 2 Liaoning Institute of Pomology, Yingkou, Liaoning, China; 3 Department of Horticulture, Faculty of Agriculture, Ataturk University, Erzurum, Türkiye

**Keywords:** anthocyanins, antioxidant, HPLC-DAD, phenolic acids, *Vaccinium corymbosum*

## Abstract

**Introduction:**

Highbush blueberries (*Vaccinium corymbosum* L.) are recognized as functional foods with exceptional health-promoting properties, yet comprehensive comparative evaluations of cultivar-dependent variation in bioactive compounds remain limited for breeding applications.

**Methods:**

This study characterized phenolic profiles, anthocyanin content, sugar composition, and antioxidant capacity of 21 highbush blueberry cultivars (19 Southern Highbush, 2 Northern Highbush) grown under uniform conditions at Liaoning Institute of Pomology, China. Phenolic compounds were quantified by HPLC-DAD, sugars were analyzed by HPLC with refractive index detection, and antioxidant activity was assessed using DPPH radical scavenging assay. Data were analyzed using ANOVA, Pearson correlation, principal component analysis (PCA), and hierarchical cluster analysis.

**Results:**

Significant cultivar effects (p<0.001) were detected for all biochemical parameters. Total phenolic content exhibited 2.7-fold variation (253–688 mg GAE/100 g DW), with 'Misty', 'Twilight', and 'Unknown' showing highest values. Total anthocyanins varied 4.4-fold (603–2678 mg/100 g DW), with 'Eureka', 'Twilight', and 'Rossini' demonstrating superior accumulation. Eight major phenolic compounds were identified, with chlorogenic acid (61.44–223.89 mg/100 g DW) predominating among phenolic acids and malvidin-3-glucoside as the principal anthocyanin. Sugar profiling revealed glucose and fructose as predominant carbohydrates (92–97% of total sugars), with total sugar content ranging 2.9-fold (6.13–18.06 g/100 g DW). DPPH radical scavenging capacity ranged from 35.26% to 69.08%, strongly correlating with total phenolic content (r=0.73). PCA explained 50.1% of cumulative variance and differentiated cultivars into four distinct metabolic clusters: sugar-anthocyanin co-accumulators ('Rossini', 'Twilight'), superior antioxidant types ('Misty', 'Magnifica', 'Unknown'), gallic acid specialists ('Jewel', 'L503', 'Ventura'), and cyanidin-3-glucoside specialist ('Eureka').

**Conclusion:**

These findings reveal extensive genotypic diversity in primary and secondary metabolism among highbush blueberry cultivars, providing valuable germplasm resources and selection criteria for breeding programs aimed at developing elite cultivars with enhanced nutritional quality and superior antioxidant properties.

## Introduction

Fruit cultivation is one of the most important branches of agriculture, providing services to humanity not only for economic benefits but also for human health and nutrition, agrotourism, and other aspects (Ercisli et al., 2007; Papp et al., 2010; Colakovic-Prguda et al., 2025). Fruits, especially berries and small shrubs, contain natural antioxidants that are essential components of a healthy diet. Numerous studies have shown that bioactive compounds such as vitamins, carotenoids, terpenes, and phenolic compounds in berries are strongly associated with the antioxidant properties obtained from their consumption. The antioxidants in berries exert their effects by scavenging reactive oxygen species, which can cause oxidative damage to cellular macromolecules and lead to tumor formation and carcinogenesis (Serce et al., 2010; Pemmari et al., 2022; Martini et al., 2023).

Blueberries (*Vaccinium* spp.), members of the Ericaceae family, have emerged as one of the most economically valuable and nutritionally significant berry crops worldwide (Wan et al., 2024; Oh et al., 2025; Tomic et al., 2025). Global blueberry production has experienced remarkable growth, more than doubling between 2010 and 2021 from 439.000 metric tons to 1.1 million metric tons, driven primarily by increasing consumer demand for health-promoting foods (Ru et al., 2024; FAOSTAT, 2023). The genus *Vaccinium* encompasses multiple species and cultivar groups, with highbush blueberries (*Vaccinium corymbosum* L.) representing the predominant cultivated type globally (Nishiyama et al., 2021). These cultivars are further categorized into northern highbush (NHB) and southern highbush (SHB) based on their chilling requirements and adaptation to different climatic conditions, with SHB cultivars developed through interspecific hybridizations between NHB and low-chill *Vaccinium* species to extend production to warmer climates (Brevis et al., 2008; Campa and Ferreira, 2018).

The remarkable health benefits associated with blueberry consumption have been extensively documented, with epidemiological studies linking regular intake to reduced risk of cardiovascular diseases, type 2 diabetes, neurodegenerative disorders, and certain cancers (Kalt et al., 2020). The growing consumer demand for blueberries reflects their recognized nutritional and functional value (Trejo-Pech et al., 2024). These protective effects are primarily attributed to the fruit’s rich composition of bioactive phytochemicals, particularly phenolic compounds, which serve as potent antioxidants capable of neutralizing reactive oxygen species (ROS) and mitigating oxidative stress-induced cellular damage (Halliwell, 1994). Among phenolic compounds, anthocyanins—the water-soluble flavonoid pigments responsible for the characteristic blue-purple coloration—have been identified as the major contributors to antioxidant activity, accounting for approximately 35-74% of total phenolic content in mature fruits (Basu and Lyons, 2012). Blueberry anthocyanins have been shown to act as powerful intracellular antioxidants in mammalian cells, with nineteen of twenty-five anthocyanin glycosides detected in human serum following consumption, directly correlated with increased serum antioxidant capacity (Kay and Holub, 2002; Bornsek et al., 2012).

The phenolic profile of blueberries comprises multiple classes of compounds including anthocyanins (primarily glycosides of delphinidin, cyanidin, petunidin, peonidin, and malvidin), hydroxycinnamic acids (notably chlorogenic acid), flavonols (quercetin derivatives), and flavan-3-ols (catechins and proanthocyanidins) (Li et al., 2016). Recent studies using ultra-performance liquid chromatography coupled with mass spectrometry (UPLC-MS) have identified up to thirty anthocyanin derivatives in blueberry cultivars, including acetylated glycosides that exhibit distinct mass fragmentation patterns (Kim et al., 2025; Wang et al., 2022). The degree of methylation and acetylation of anthocyanins varies considerably among cultivars, potentially influencing both stability and bioactivity (Wang et al., 2022).

Despite extensive research on blueberry phytochemistry, comprehensive and integrated evaluations of cultivar-dependent variation in phenolic composition, sugar metabolism, and antioxidant capacity across large panels of highbush blueberry cultivars remain limited. Previous investigations have demonstrated significant inter-cultivar differences in total anthocyanin content (0.74-4.27 mg cyanidin-3-glucoside equivalents/g fresh weight), total phenolic content (0.77-3.69 mg gallic acid equivalents/g fresh weight), and antioxidant activity (0.7-2.1 mg quercetin equivalents/g fresh berries), with these variations attributed to genetic factors, environmental conditions, and cultivation practices (Kim et al., 2013; Shibata et al., 2021). Notably, rabbiteye blueberries have been reported to contain significantly higher anthocyanin and phenolic contents compared to highbush cultivars, suggesting substantial interspecific variation (Shibata et al., 2021). Production system also significantly influences phenolic characteristics, with non-heated greenhouse and open field systems generally yielding higher phenolic content and antioxidant capacity compared to heated greenhouse systems (Jung et al., 2021). Furthermore, while strong correlations between total phenolic content and antioxidant activity have frequently been reported (Li et al., 2013), recent evidence suggests that antioxidant efficacy depends not only on total concentration but also on the specific composition and relative proportions of individual phenolic compounds (Mengist et al., 2020). Genetic diversity among blueberry cultivars has been reported to be closely associated with variation in anthocyanin accumulation and phenolic composition (Li et al., 2018).

Understanding genotype-dependent variations in both sugar and phenolic composition provides a comprehensive assessment of fruit quality, enabling identification of superior genotypes that combine excellent nutritional properties with desirable sensory (Nawaz et al., 2024; Ozkan et al., 2025). For breeders, such knowledge facilitates selection of genotypes with superior nutritional profiles and desirable taste attributes. For growers, it enables informed cultivar selection based on target markets, intended applications (fresh market versus processing), and regional growing conditions. For consumers and nutritionists, it provides evidence-based guidance for maximizing both health benefits and eating quality through strategic fruit selection.

Therefore, a clear knowledge gap exists in the integrated characterization of sugars, individual phenolic compounds, total phenolics, anthocyanins, and antioxidant capacity across diverse highbush blueberry germplasm using multivariate analytical approaches.

Therefore, the present study was undertaken with the following objectives: (1) to comprehensively characterize the phenolic composition and sugar content of 21 highbush blueberry cultivars (19 southern highbush and 2 northern highbush) grown under uniform environmental conditions using HPLC-DAD analysis, (2) to quantify total phenolic content, total anthocyanin content, soluble sugars, and antioxidant capacity (DPPH assay) with triplicate measurements to ensure statistical reliability, (3) to identify cultivar-specific variations and patterns in phytochemical and sugar profiles, (4) to evaluate correlations between phenolic compounds, sugar content, and antioxidant activity, and (5) to identify superior genotypes combining high phytochemical content with desirable sugar profiles for breeding programs and functional food development. This study provides one of the most comprehensive metabolite-based evaluations of highbush blueberry germplasm reported to date, offering valuable insights for integrated breeding strategies targeting multiple quality traits.

## Materials and methods

### Plant material

A total of 21 highbush blueberry (*Vaccinium corymbosum* L.) cultivars were used in this investigation during the 2024 growing season. All cultivars were cultivated under controlled greenhouse conditions at the Small Fruits Demonstration Area of the Liaoning Institute of Pomology, located in Yingkou City, Liaoning Province, China (40°40′N, 122°14′E) (**Table 1**). The greenhouse facilities were equipped with automated fertigation systems, and all plants were maintained under uniform irrigation, fertilization, and management practices to ensure consistent growing conditions. For each cultivar, fruits were harvested from five plants at the commercial ripening stage specific to each cultivar, as harvest dates differed according to individual ripening time. Sampling was performed using three biological replicates per cultivar, with each replicate consisting of an equal mass of fruits. Harvesting was conducted during the early morning hours to minimize temperature-related variation. Only fully ripe fruits with uniform color and size and free from visible defects were selected. After harvest, fruit samples were immediately frozen, freeze-dried, and finely ground into powder. The dried samples were then transported under controlled conditions to the Department of Horticulture, Faculty of Agriculture, Çukurova University, Adana, Turkey, where all biochemical analyses were performed.

**Table 1:** List of highbush blueberry cultivars used in phenolic compound analysis.

No.	Cultivars name	Type
1	Jewel	Southern Highbush
2	Camellia	Southern Highbush
3	Misty	Southern Highbush
4	Unknown	Southern Highbush
5	Magnifica	Southern Highbush
6	Suziblue	Southern Highbush
7	Windsor	Southern Highbush
8	Rossini	Southern Highbush
9	Eureka Sunrise	Southern Highbush
10	Twilight	Southern Highbush
11	Springhigh	Southern Highbush
12	Scintilla	Southern Highbush
13	Meadowlark	Southern Highbush
14	Star	Southern Highbush
15	Ventura	Southern Highbush
16	Rocio	Southern Highbush
17	Farthing	Southern Highbush
18	L503	Northern Highbush
19	Emerald	Southern Highbush
20	Eureka	Southern Highbush
21	Legacy	Northern Highbush

### Chemicals

A comprehensive array of chemical compounds and reagents were employed in this study. These included Folin-Ciocalteu reagent, along with sodium carbonate, acetonitrile, potassium peroxide sulfate, and various solvents such as ethanol and methanol. Additional materials consisted of calcium chloride, aluminum chloride, potassium chloride, sodium acetate, sodium nitrite, and sodium hydroxide. Standard compounds utilized comprised gallic acid, myricetin, caffeic acid, p-coumaric acid, ellagic acid, quercetin, kaempferol, catechin, chlorogenic acid, and cyanidin-3-glucoside. The antioxidant assays employed Trolox and 2, 2-diphenyl-1-picrylhydrazyl (DPPH). All chemical reagents and reference standards, including gallic acid, chlorogenic acid, catechin, DPPH, and Folin-Ciocalteu reagent, were sourced from Sigma-Aldrich (St. Louis, MO, USA). Solvents used throughout the experiments were of high-performance liquid chromatography (HPLC) grade quality, obtained from Merck (Darmstadt, Germany).

### Determination of antioxidant activity and free radical scavenging

#### DPPH radical scavenging activity

The antioxidant capacity and free radical scavenging potential of dried blueberry fruit extracts were evaluated using the DPPH assay following the methodology of Pękal and Pyrzynska (2013). The assay protocol involved mixing 0.1 mL of sample extract with 2.4 mL of methanolic DPPH solution (9 × 10^-5^ mol/L). Following thorough mixing, the reaction mixture was maintained at ambient temperature in darkness for 30 minutes, after which spectrophotometric absorbance was recorded at 518 nm using a blank as reference. The Radical Scavenging Activity (DPPH_RSA) was calculated as a percentage using the following equation:


$$DPPH\;RSA\;(\%)=(\frac{A\;blank-\;\;A\;sample\;\;}{A\;blank})\times 100$$

where A_control represents the absorbance of DPPH solution without extract, and A_sample denotes the absorbance of the extract-DPPH reaction mixture.

### Preparation of blueberry extracts

Phenolic compounds were extracted using a solvent system optimized for blueberry anthocyanins: methanol: water:trifluoroacetic acid (70:30:1, v/v/v) as described by Barnes et al. (2009). Briefly, 2 mL of the homogenized blueberry sample was mixed with 3 mL of 70% methanol containing 0.1% trifluoroacetic acid, sonicated for 20 minutes, and centrifuged at 5, 500 rpm for 15 minutes. The supernatant was then filtered through a 0.45 µm membrane and used for HPLC analysis.

### Determination of total phenolics

The quantification of total phenolic compounds in the extracts was performed using the Folin-Ciocalteu (FC) colorimetric assay as described by Akbari et al. (2023). The analytical procedure involved mixing 0.1 mL of sample extract with 0.1 mL of FC reagent and 0.9 mL of distilled water, allowing the mixture to react for 5 minutes. Subsequently, 1 mL of 7% (w/v) sodium carbonate (Na_2_CO_3_ solution and 0.4 mL of water were added, and the mixture was incubated for an additional 30 minutes. Following incubation, the absorbance was recorded at 765 nm using a spectrophotometer. A blank solution containing water and reagents without extract was used as the reference. The total phenolic content was calculated using a gallic acid standard curve and expressed as milligrams of gallic acid equivalents (GAE) per 100 grams of dried fruit weight. Each sample was analyzed in triplicate to ensure reproducibility.

### Determination of total monomeric anthocyanins

The quantification of total monomeric anthocyanins was conducted employing the pH-differential spectrophotometric method as outlined by Lee et al. (2005). The analytical procedure involved dividing 1 mL of sample extract between two separate volumetric flasks. The first flask was brought to volume with potassium chloride (KCl) buffer at pH 1.0, while the second flask was brought to volume with sodium acetate (CH_3_COONa) buffer at pH 4.5. Both buffer solutions were allowed to equilibrate at ambient temperature for 30 minutes. Following equilibration, spectrophotometric measurements were taken at wavelengths of 520 nm and 700 nm for each buffer solution. Anthocyanin concentration was calculated and expressed as milligrams of cyanidin-3-glucoside (C3G) equivalents per 100 grams of sample dry weight. All measurements were conducted in triplicate for analytical precision.

### Sugars determination

The quantification of sucrose, glucose, fructose, and total sugar levels was performed using high-performance liquid chromatography (Shimadzu LC 20A VP, Kyoto, Japan) equipped with a refractive index detector. Chromatographic separation was achieved employing a reverse-phase Ultrasphere Coregel-87 C column (300 mm × 7.8 mm, 5 µm particle size) maintained at 70°C. The mobile phase consisted of ultrapure water delivered at a flow rate of 0.6 mL/min under isocratic elution conditions. Each sample injection volume was 20 µL. Sugar concentrations were determined using external standard calibration curves and reported as percentage of dry weight (DW).

### Analysis of the phenolic compounds using HPLC

Phenolic compounds were analyzed by high-performance liquid chromatography (HPLC) based on the method described by Flandez et al. (2023), with slight modifications. The analysis was conducted using an Agilent Eclipse XDB-C18 column (250 × 4.6 mm, 5 µm) maintained at 30°C. A binary solvent system was used: solvent A (2.5% formic acid in water) and solvent B (2.5% formic acid in acetonitrile), applied in gradient mode at a flow rate of 1.0 mL min^-^¹. The gradient program was as follows: 0–5 min, 95% A; 5–15 min, 95–80% A; 15–30 min, 80–60% A; 30–40 min, 60–40% A; 40–45 min, 40–95% A, followed by 5 min of re-equilibration under the initial conditions. Detection was performed at 260, 320, and 360 nm using a diode array detector (DAD). Samples were filtered through 0.45 µm syringe filters before injection, and 20 µL of each sample was injected for analysis.

All phenolic compounds were identified and quantified by comparing retention times (± 0.1 min) and UV–Vis absorbance spectra obtained by DAD with those of authentic reference standards. The standards used included gallic acid, caffeic acid, p-coumaric acid, chlorogenic acid, ellagic acid, myricetin, quercetin, kaempferol, catechin, and others, all purchased from Sigma-Aldrich (St. Louis, MO, USA). Calibration curves for each compound were constructed using five concentrations prepared from 1 mg mL^-^¹ stock solutions in methanol. Each injection was performed in duplicate as technical replicates, and the mean values were used for quantification.

### Statistical analysis

All statistical analyses were performed using R software (version 4.4.2). One-way ANOVA was used to assess significant differences among cultivars for all biochemical traits, and mean separation was conducted using Tukey’s HSD test at p ≤ 0.05. Pearson correlation coefficients among phenolic compounds, anthocyanins, sugars, and antioxidant parameters were calculated using the corrplot package.

Principal Component Analysis (PCA) was performed using the FactoMineR and factoextra packages to identify multivariate patterns among cultivars. Hierarchical clustering and heatmap visualization were generated using pheatmap based on Z-score standardized data. Bubble plots, correlation matrices, and sugar composition graphs were produced using ggplot2. All metabolite data were expressed as mean±SE.

## Results and discussion

### Bioactive compounds

Analysis of variance revealed highly significant differences (p<0.001) among the 21 highbush blueberry cultivars for all measured phytochemical parameters, demonstrating substantial genetic variation within commercial germplasm. The cultivar panel comprised 19 Southern Highbush and 2 Northern Highbush varieties, enabling comprehensive assessment of genotypic diversity.

### Total phenolic content

**Figure 1:**
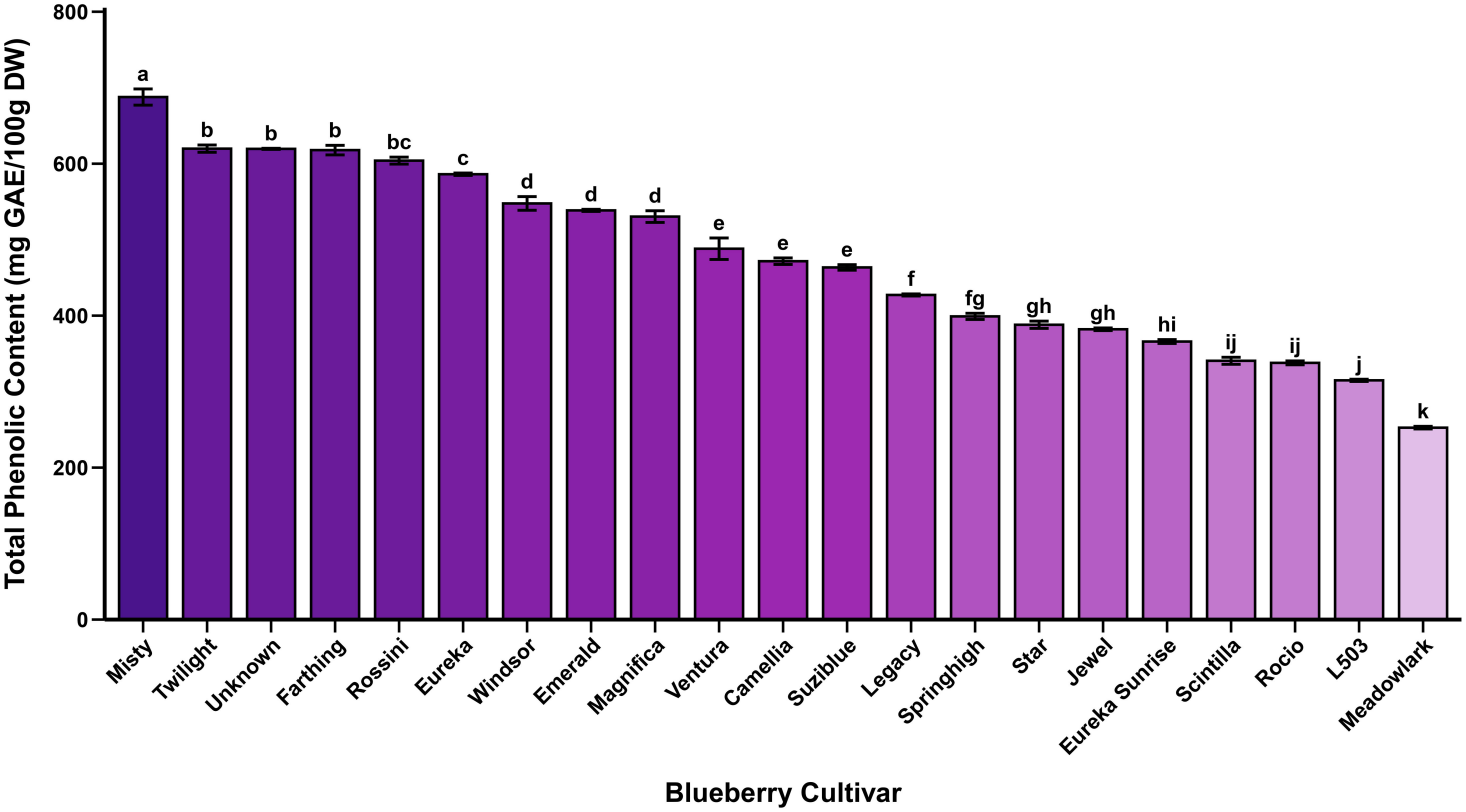
Comparison of total phenolic content among highbush blueberry cultivars. Values are mean±SE (n=3). Different letters denote significant differences at p<0.05 (Tukey’s HSD test). GAE: gallic acid equivalents.

Total phenolic content (TPC) exhibited substantial cultivar-dependent variation, ranging from 253 mg GAE/100g DW in ‘Meadowlark’ to 688 mg GAE/100 g DW in ‘Misty’, representing a 2.7-fold difference among cultivars (**Table 2**, **Figure 1**). The Southern Highbush cultivar ‘Misty’ demonstrated the highest total phenolic accumulation, followed by ‘Twilight’ (619.80 mg GAE/100g DW), ‘Unknown’ (619.52 mg GAE/100 g DW), and ‘Farthing’ (617.85 mg GAE/100 g DW). The Northern Highbush cultivar ‘Legacy’ exhibited moderate total phenolic content (427 mg GAE/100 g DW), while ‘L503’ displayed the lowest value among Northern Highbush cultivars at 315 mg GAE/100 g DW. The mean TPC across all 21 cultivars was 468 mg GAE/100 g DW. These results are consistent with previous reports on highbush blueberry germplasm. Connor et al. (2002) documented significant genotypic effects on total phenolic content among 16 highbush cultivars. Similarly, Kim et al. (2013) reported substantial variation in TPC among 45 blueberry cultivars grown in Korea. Our results align with the range reported by Dragović-Uzelac et al. (2010), who observed total phenolic contents between 274 and 694 mg GAE/100 g in Croatian-grown cultivars, and are within the ranges documented in recent studies (Pavliuk et al., 2025; Lachowicz-Wiśniewska et al., 2024).

**Table 2:** Mean comparison of antioxidant activity and bioactive compound content in dried highbush blueberry cultivars.

Cultivar	DPPH radical scavenging (%)	Total phenol (mg/100g GAE)	Total anthocyanin (mg/100g)
Camellia	62.15±0.35 cde	471.66±4.32 e	961.16±11.91 i
Emerald	67.18±0.25 ab	538.52±1.41 d	1813.58±29.30 c
Eureka	67.97±0.48 a	586.11±1.60 c	2678.12±33.91 a
Eureka Sunrise	55.30±0.90 g	365.90±2.58 hi	1144.47±2.29 h
Farthing	69.08±0.43 a	617.85±6.31 b	1634.98±8.15 d
Jewel	62.53±1.41 cd	382.06±1.75 gh	1186.06±10.62 gh
L503	54.92±0.69 gh	314.84±1.48 j	603.16±4.84 k
Legacy	48.45±0.34 i	427.21±1.38 f	1582.26±25.33 de
Magnifica	62.99±0.89 bcd	530.36±7.69 d	1409.57±25.14 f
Meadowlark	35.26±0.82 k	252.56±1.74 k	765.12±5.32 j
Misty	69.03±0.65 a	687.71±10.66 a	1651.91±11.17 d
Rocio	57.87±1.42 efg	337.84±2.57 ij	885.49±14.13 i
Rossini	65.09±0.60 abc	604.00±4.62 bc	2124.38±20.69 b
Scintilla	50.77±0.81 hi	340.58±4.64 ij	887.09±14.78 i
Springhigh	56.79±1.31 fg	399.02±4.08 fg	1126.43±9.06 h
Star	56.56±0.14 fg	388.01±4.79 gh	1141.06±27.70 h
Suziblue	60.04±0.84 def	463.45±3.64 e	1097.17±11.45 h
Twilight	67.78±0.80 a	619.80±4.79 b	2169.48±27.82 b
Unknown	65.31±0.84 abc	619.52±0.73 b	1243.29±9.06 g
Ventura	43.83±0.86 j	488.08±14.14 e	1514.30±11.62 e
Windsor	67.88±0.68 a	547.68±8.99 d	1487.84±17.69 e

The substantial genotypic variation observed in TPC suggests cultivar-specific differences in phenylpropanoid metabolism and carbon partitioning between primary and secondary metabolites. Southern Highbush cultivars, developed through interspecific hybridization of Northern Highbush (*Vaccinium corymbosum*) with various southern species including V. darrowii and V. ashei, often exhibit distinct phytochemical profiles compared to their Northern Highbush counterparts (Lobos and Hancock, 2015).

### DPPH radical scavenging activity and antioxidant capacity

**Figure 2:**
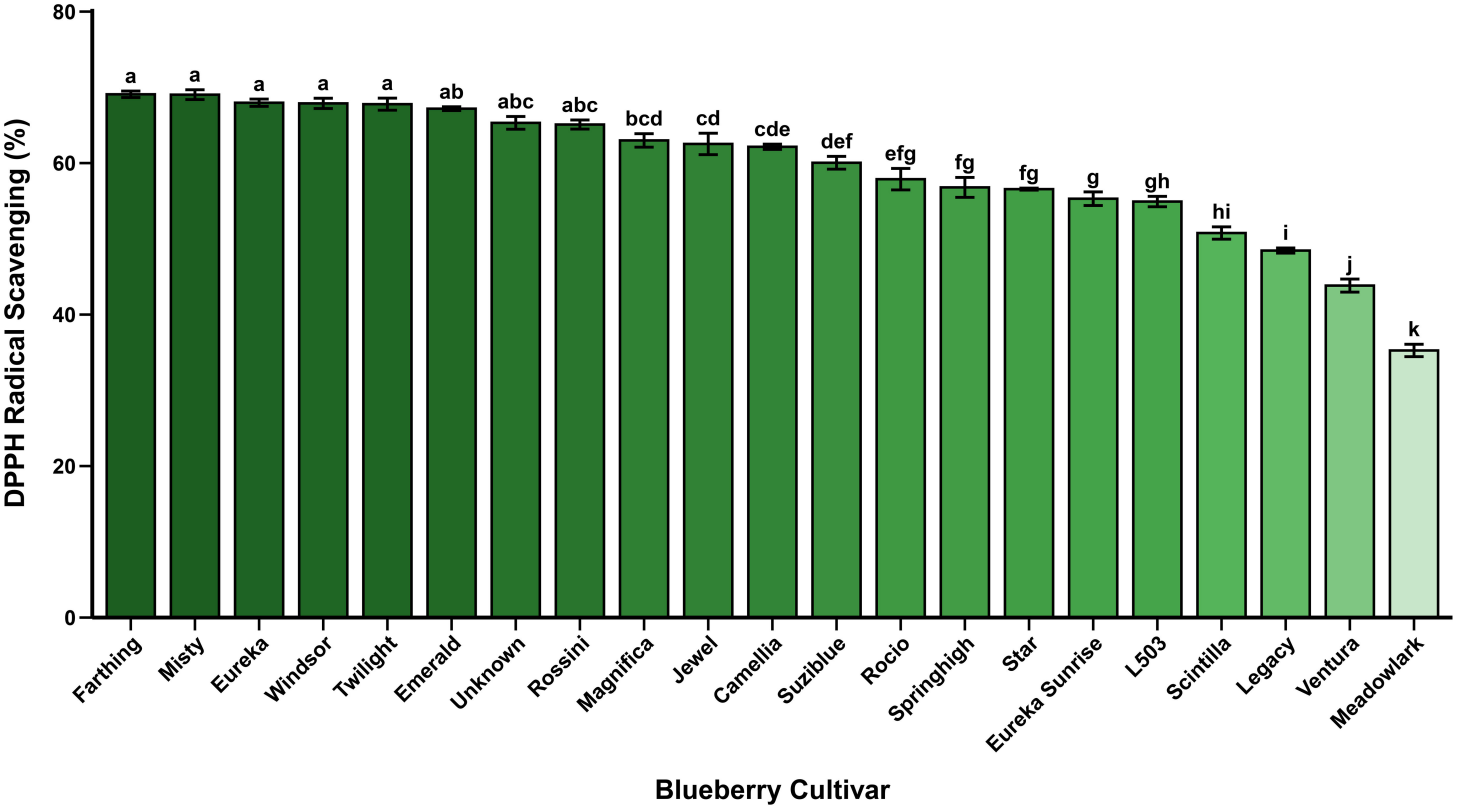
Comparison of DPPH radical scavenging capacity among dried blueberry cultivars. Values are mean±SE (n=3). Different letters denote significant differences at p<0.05 (Tukey’s HSD test). DPPH: 2, 2-diphenyl-1-picrylhydrazyl.

DPPH radical scavenging capacity among dried highbush blueberry cultivars ranged from 35.26±0.82% in ‘Meadowlark’ to 69.08±0.43% in ‘Farthing’, representing a 2.0-fold variation (**Table 2****;****Figure 2**). The cultivar ‘Farthing’ exhibited the highest antioxidant capacity, followed by ‘Misty’ (69.03±0.65%), ‘Eureka’ (67.97±0.48%), ‘Windsor’ (67.88±0.68%), and ‘Twilight’ (67.78±0.80%). Conversely, cultivars with the lowest antioxidant activity included ‘Meadowlark’ (35.26±0.82%), ‘Ventura’ (43.83±0.86%), ‘Legacy’ (48.45±0.34%), ‘Scintilla’ (50.77±0.81%), and ‘L503’ (54.92±0.69%). The mean DPPH radical scavenging capacity across all 21 cultivars was 59.78%, highlighting substantial genotypic variation in antioxidant performance (**Table 2**).

The antioxidant capacity of blueberry fruit is primarily attributed to phenolic compounds, particularly anthocyanins, flavonols, hydroxycinnamic acids, and proanthocyanidins, which function through multiple mechanisms including hydrogen atom transfer (HAT), single electron transfer (SET), and transition metal chelation (Zeb, 2020). The DPPH assay, which operates predominantly through SET-based mechanisms, measures the ability of antioxidants to donate electrons or hydrogen atoms to stabilize the nitrogen-centered DPPH radical (Apak et al., 2016), reflecting the total reducing capacity of phenolic metabolites. The superior antioxidant activity observed in ‘Farthing’, ‘Misty’, and ‘Eureka’ correlates strongly with their elevated total phenolic and anthocyanin contents (**Table 2**), demonstrating the direct contribution of secondary metabolite accumulation to radical scavenging capacity. The synergistic interactions among different phenolic classes, including copigmentation effects and antioxidant regeneration cycles, further enhance the overall antioxidant potential of these elite cultivars.

Our findings align with previous research demonstrating strong correlations between phenolic content and antioxidant capacity in blueberry cultivars. Connor et al. (2002) evaluated genotypic and environmental variation in antioxidant activity among 16 highbush blueberry cultivars across multiple locations and reported that cultivar effects accounted for the majority of variation, with total phenolic and anthocyanin contents being strongly correlated with antioxidant activity. Similarly, Skrovankova et al. (2015) compared antioxidant activities across different berry types and found that blueberries exhibited superior radical scavenging capacity attributable to their unique phenolic composition, particularly the high concentration of anthocyanins and hydroxycinnamic acid derivatives. The correlation between phenolic content and DPPH scavenging activity was further confirmed by Kraujalytė et al. (2015), who demonstrated that commercial and wild blueberry genotypes with elevated total phenolic content consistently showed higher antioxidant capacity across multiple assay systems, including DPPH, FRAP, and ABTS methods.

### Total anthocyanin content

**Figure 3:**
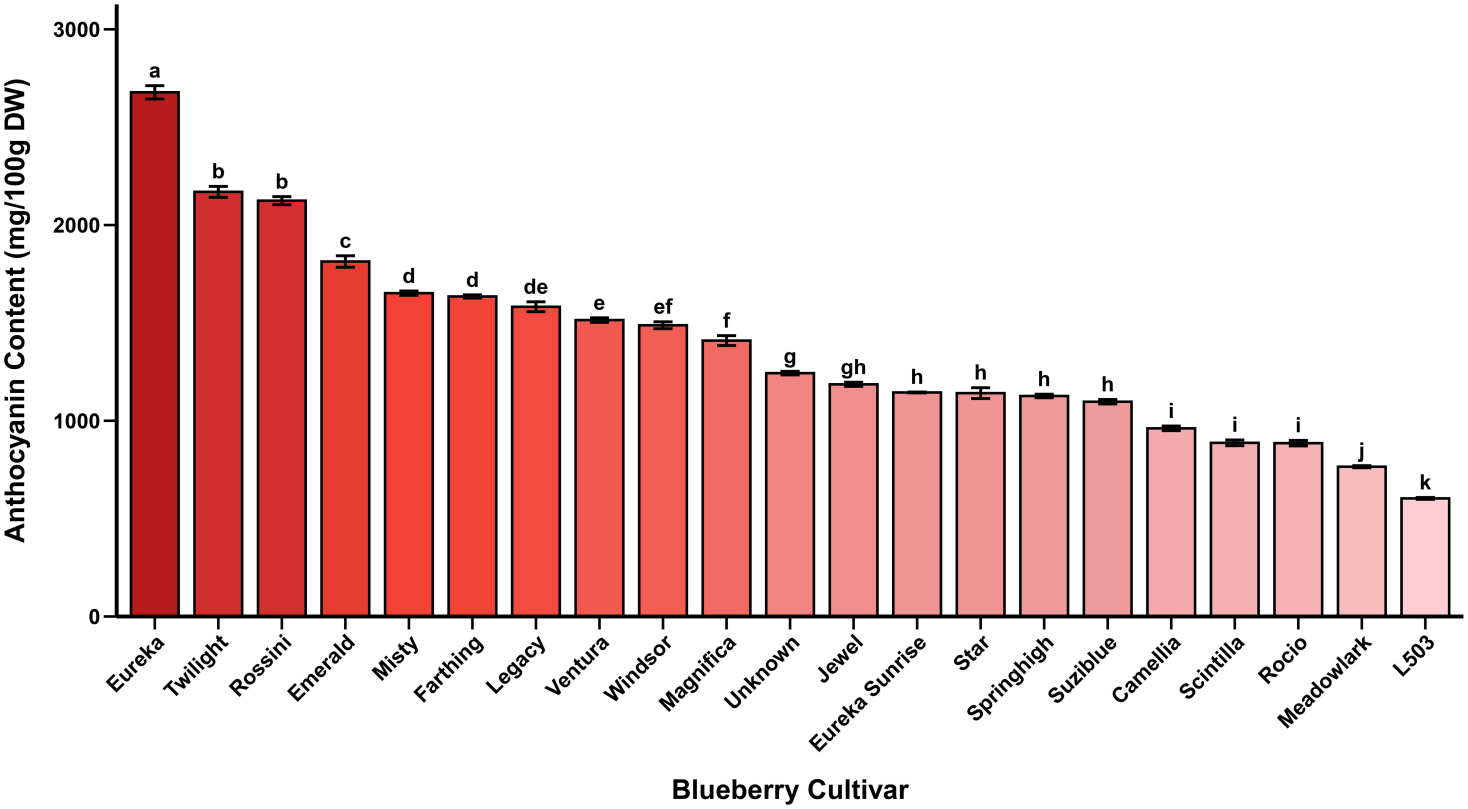
Comparison of anthocyanin content among dried blueberry cultivars. Values are mean±SE (n=3). Different letters denote significant differences at p<0.05 (Tukey’s HSD test).

Anthocyanin content in dried highbush blueberry cultivars exhibited remarkable genotypic variation, ranging from 603 mg/100 g in ‘L503’ to 2678 mg/100 g in ‘Eureka’, representing a 4.4-fold difference among cultivars. The cultivar ‘Eureka’ demonstrated the highest anthocyanin accumulation, followed by ‘Twilight’ (2169 mg/100 g), ‘Rossini’ (2124 mg/100 g), ‘Emerald’ (1813 mg/100 g), and ‘Misty’ (1651 mg/100 g). Conversely, cultivars with the lowest anthocyanin content included ‘L503’ (603 mg/100 g), ‘Meadowlark’ (765 mg/100 g), ‘Rocio’ (885 mg/100 g), ‘Scintilla’ (887 mg/100 g), and ‘Camellia’ (961 mg/100 g). The mean anthocyanin content across all 21 cultivars was 1357 mg/100 g, expressed as cyanidin-3-glucoside equivalents. These results are summarized in **Table 2** and illustrated in **Figure 3**.

Anthocyanin biosynthesis is governed by a complex network of structural and regulatory components within the flavonoid branch of the phenylpropanoid pathway (Song et al., 2023; Nguyen et al., 2023). In Vaccinium species, anthocyanins are formed through a series of enzymatic reactions leading to the production of stable glycosylated pigments, which are subsequently transported and stored in the vacuole (Lafferty et al., 2022). The pronounced genotypic variation observed in the present study may therefore reflect cultivar-dependent differences in the overall regulation and efficiency of anthocyanin biosynthetic and stabilization processes, rather than direct evidence of specific gene expression or enzyme activity.

Cultivars exhibiting superior anthocyanin accumulation, such as ‘Eureka’, ‘Twilight’, and ‘Rossini’, may possess a greater metabolic capacity for anthocyanin production, vacuolar sequestration, and pigment stabilization. In addition, differences in pH-dependent stability and copigmentation interactions with other phenolic compounds may further contribute to the observed diversity in anthocyanin content among cultivars. However, detailed molecular and enzymatic analyses would be required to confirm the underlying regulatory mechanisms responsible for these differences.

Our results are consistent with previous studies documenting extensive cultivar-dependent variation in blueberry anthocyanin profiles. Chai et al. (2021) analyzed 74 blueberry cultivars from China and identified malvidin-3-O-galactoside, delphinidin-3-O-galactoside, and malvidin-3-O-glucoside as the predominant anthocyanins. Similarly, Kim et al. (2013) evaluated 45 northern highbush and half-highbush blueberry cultivars grown in Korea and reported anthocyanin contents ranging from 167 to 677 mg cyanidin-3-glucoside per 100 g fresh weight. The variation observed in our study is comparable to that reported by Li et al. (2017), who demonstrated that anthocyanin concentrations in commercial blueberry cultivars are primarily genotype-dependent rather than solely influenced by environmental factors. The higher absolute values observed in the present study reflect the concentration effect associated with the use of dried samples.

### Sugar composition

Analysis of variance revealed highly significant differences (*p*<0.01) among the 21 highbush blueberry cultivars for all individual sugar components (sucrose, glucose, fructose) and total sugar content measured on a dry weight basis. These significant cultivar effects underscore the substantial genetic diversity in carbohydrate metabolism and accumulation capacity present in the evaluated germplasm, providing valuable resources for breeding programs and cultivar selection strategies.

**Figure 4:**
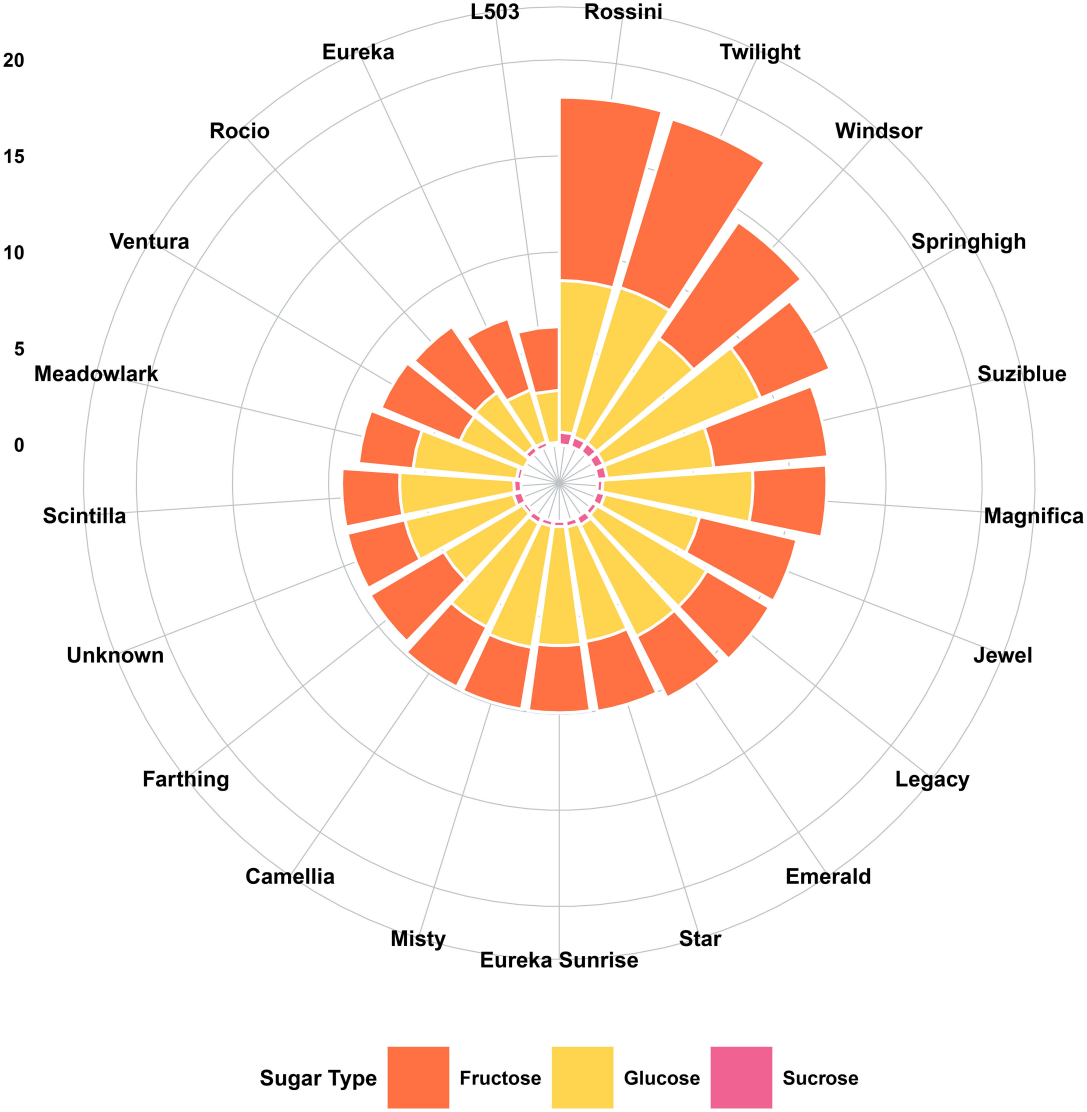
Radial visualization of sugar composition in 21 highbush blueberry cultivars expressed on a dry weight basis.

Comprehensive soluble sugar profiling on a dry weight basis revealed that glucose and fructose constituted the predominant carbohydrates across all 21 cultivars examined (**Table 3**, **Figure 4**). Glucose concentrations ranged from 2.70 g/100 g DW (‘L503’) to 8.85 g/100 g DW (‘Springhigh’), exhibiting a 3.3-fold variation among genotypes. Fructose content demonstrated comparable genetic diversity, spanning from 2.85 g/100 g DW (‘Meadowlark’) to 9.53 g/100 g DW (‘Rossini’), also representing a 3.3-fold range. These findings are in complete accordance with Cvetković et al. (2022) and Akšić et al. (2019), who established that glucose and fructose consistently constitute 92-97% of the total soluble sugar pool in *Vaccinium corymbosum* L. fruits. This hexose-dominant sugar profile distinguishes blueberries from other berry crops and directly influences their metabolic, sensory, and nutritional properties.

The glucose-to-fructose ratio across cultivars approximated unity (mean G/F = 1.03, range 0.95-1.15) (**Table 3**), indicating nearly equimolar concentrations of these two hexose monosaccharides in mature fruits (**Figure 4**). This balanced ratio is a hallmark of ripe Vaccinium species and reflects the enzymatic hydrolysis of sucrose during fruit development. Li et al. (2020) demonstrated that this approximate 1:1 equilibrium between glucose and fructose is established early in fruit development and maintained throughout ripening. The physiological significance of this ratio extends beyond metabolism; fructose exhibits a relative sweetness coefficient of approximately 1.2-1.8 relative to sucrose, and roughly 1.7-2.0 times sweeter than glucose (Fontvieille et al., 1989), thereby contributing disproportionately to perceived sweetness despite equivalent concentrations. Consequently, cultivars with slightly elevated fructose-to-glucose ratios may deliver enhanced sweetness perception at identical total sugar levels, as demonstrated by sensory evaluation studies conducted by Gilbert et al. (2015).

**Table 3:** Sugar composition (g/100 g DW) in fruits of 21 highbush blueberry cultivars.

Cultivar	Sucrose	Glucose	Fructose	Total sugar
Rossini	0.66±0.01 a	7.87±0.03 b	9.53±0.01 a	18.06±0.05 a
Twilight	0.54±0.00 b	8.12±0.04 b	9.16±0.23 a	17.82±0.18 a
Windsor	0.55±0.01 b	6.59±0.09 c	7.33±0.13 b	14.48±0.24 b
Springhigh	0.52±0.00 bc	8.85±0.03 a	3.92±0.09 efg	13.29±0.12 c
Suziblue	0.51±0.00 bcd	5.58±0.00 gh	5.96±0.03 c	12.04±0.04 d
Magnifica	0.28±0.01 hij	7.80±0.02 b	3.84±0.17 efgh	11.93±0.14 d
Jewel	0.46±0.00 de	5.05±0.06 ij	5.21±0.04 cd	10.71±0.11 e
Legacy	0.30±0.00 ghi	6.62±0.05 c	3.78±0.07 efgh	10.70±0.03 e
Emerald	0.44±0.02 e	6.47±0.10 cd	3.58±0.11 fghi	10.50±0.24 e
Star	0.32±0.01 fghi	6.03±0.03 ef	3.68±0.04 efghi	10.02±0.08 ef
Eureka Sunrise	0.28±0.00 hij	6.17±0.01 def	3.49±0.25 fghi	9.93±0.25 ef
Misty	0.28±0.01 hij	6.38±0.04 cde	3.26±0.09 ghi	9.91±0.04 ef
Camellia	0.35±0.00 fg	6.02±0.05 ef	3.49±0.00 fghi	9.87±0.06 ef
Farthing	0.22±0.01 j	4.77±0.06 j	4.45±0.06 de	9.44±0.01 fg
Unknown	0.47±0.00 cde	5.79±0.06 fgh	3.09±0.31 ghi	9.34±0.37 fg
Scintilla	0.38±0.00 f	5.92±0.00 fg	3.02±0.08 hi	9.32±0.08 fg
Meadowlark	0.24±0.02 j	5.41±0.04 hi	2.85±0.33 i	8.50±0.27 gh
Ventura	0.00±0.00 l	3.62±0.22 k	4.46±0.17 de	8.07±0.40 h
Rocio	0.33±0.01 fgh	3.40±0.10 k	4.15±0.10 ef	7.88±0.22 hi
Eureka	0.26±0.00 ij	2.81±0.08 l	3.88±0.01 efg	6.96±0.09 ij
L503	0.14±0.01 k	2.70±0.01 l	3.30±0.01 ghi	6.13±0.02 j

Data are presented as means±SE (n = 3). Values in the same column followed by different letters are significantly different at P < 0.05 level (Duncan's multiple range test). Total sugars = sucrose + glucose + fructose. Sugars were quantified by HPLC. DW = dry weight.

A distinguishing biochemical characteristic of all examined cultivars was the remarkably low sucrose concentration, which ranged from complete absence (0.00 g/100 g DW in ‘Ventura’) to a maximum of only 0.66 g/100 g DW in ‘Rossini’ (**Table 2**, **Figure 4**). Across the 21 cultivars, sucrose represented merely 0-3.7% of total soluble sugars, contrasting sharply with strawberries where sucrose can comprise 30-70% of the total sugar pool (Akšić et al., 2019). The cultivars with minimal sucrose accumulation included ‘Ventura’ (non-detectable), ‘L503’ (0.14 g/100 g DW), and ‘Farthing’ (0.22 g/100 g DW), whereas those with relatively higher concentrations comprised ‘Rossini’ (0.66 g/100 g DW), ‘Windsor’ (0.55 g/100 g DW), and ‘Twilight’ (0.54 g/100 g DW). Despite these absolute differences among cultivars being statistically significant (*p*<0.05), all values remained within an exceptionally narrow range compared to most fruit species (**Table 3**, **Figure 4**).

Total soluble sugar content on a dry weight basis, calculated as the sum of sucrose, glucose, and fructose, exhibited substantial cultivar-dependent variation ranging from 6.13 g/100 g DW (‘L503’) to 18.06 g/100 g DW (‘Rossini’), representing a 2.9-fold difference between extremes (**Table 2**, **Figure 4**). Based on total sugar accumulation capacity, cultivars were stratified into three distinct phenotypic classes: (1) High-sugar cultivars (>15.0 g/100 g DW) comprised ‘Rossini’ (18.06), ‘Twilight’ (17.82), and ‘Windsor’ (14.48); (2) Intermediate-sugar cultivars (9.5-15.0 g/100 g DW) included ‘Springhigh’ (13.29), ‘Suziblue’ (12.04), ‘Magnifica’ (11.93), ‘Jewel’ (10.71), ‘Legacy’ (10.70), ‘Emerald’ (10.50), ‘Star’ (10.02), ‘Eureka Sunrise’ (9.93), and ‘Misty’ (9.91); and (3) Low-sugar cultivars (<9.5 g/100 g DW) encompassing ‘Camellia’ (9.87), ‘Farthing’ (9.44), ‘Unknown’ (9.34), ‘Scintilla’ (9.32), ‘Meadowlark’ (8.50), ‘Ventura’ (8.07), ‘Rocio’ (7.88), ‘Eureka’ (6.96), and ‘L503’ (6.13). All differences among cultivars were statistically significant at *p*<0.05 according to Duncan’s multiple range test, as illustrated in **Figure 4**.

The total sugar content range documented in this investigation on a dry weight basis is consistent with previously published reports on highbush blueberry germplasm. Cvetković et al. (2022) reported total sugars ranging from 56.72 to 65.13 g/kg DW (5.67-6.51 g/100 g DW) in cultivars Chandler, Bluecrop, and Duke at commercial harvest maturity, corresponding to the lower quartile of our observed range.

Zhang et al. (2020) documented significant regional variation in sugar content among 11 blueberry cultivars grown in two provinces of northern China, with total sugars ranging from 7.8 to 15.2 g/100 g DW. Their study further demonstrated that genetic (cultivar) effects were greater than environmental (location) effects for sugar accumulation, emphasizing the predominant role of genetic factors in determining fruit carbohydrate profiles. The comprehensive range of sugar contents observed in our 21-cultivar collection (6.13-18.06 g/100 g DW) encompasses and extends beyond these previous reports, suggesting that the evaluated germplasm represents the full spectrum of natural genetic variation for sugar accumulation in highbush blueberry.

The highly significant (p<0.05) genetic variation in sugar composition provides critical information for multiple applications. For fresh market production, cultivars with high total sugar and elevated fructose ratios—particularly ‘Rossini’, ‘Twilight’, and ‘Windsor’-represent optimal selections, aligning with consumer preference criteria (Gilbert et al., 2015).

### Individual phenolic compounds

**Figure 5:**
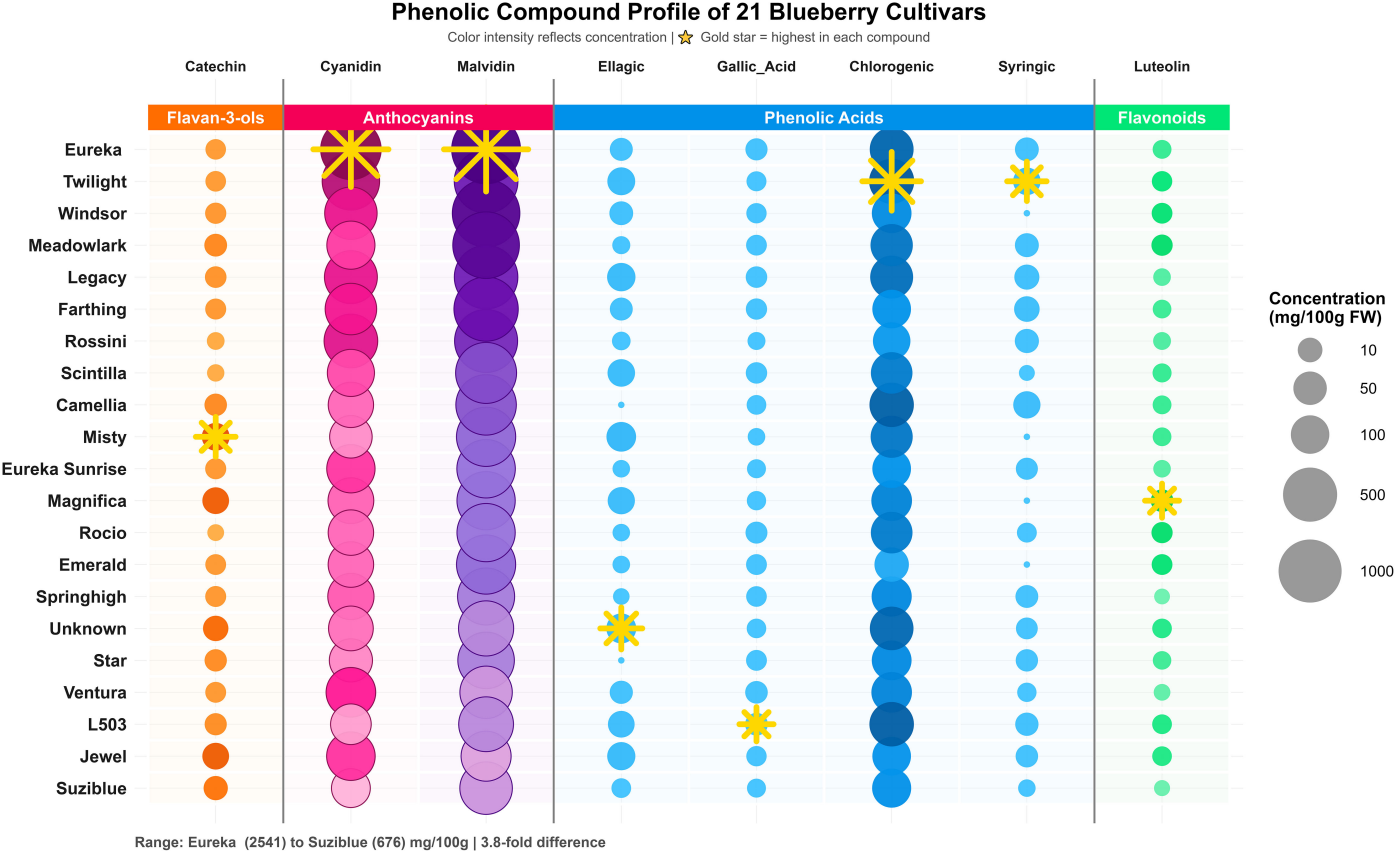
Phenolic compound and anthocyanin profiles of 21 blueberry cultivars. Bubble size represents concentration (mg/100g DW), color intensity shows relative levels, and stars mark highest values.

All cultivars differed significantly at the 1% level (*p*<0.01) for individual phenolic compound concentrations. Although the ANOVA table is not included in the text, results are summarized in **Table 4** and illustrated in **Figure 5**. The comprehensive phenolic profiling revealed eight major compounds across the 21 blueberry cultivars: chlorogenic acid, gallic acid, syringic acid, and ellagic acid representing phenolic acids; cyanidin-3-glucoside and malvidin-3-glucoside as anthocyanins; catechin as a flavan-3-ol; and luteolin as a flavonoid.

**Table 4:** Mean comparison of anthocyanin and phenolic compounds (mg/100 g dry weight) in fruits of 21 blueberry cultivars.

Cultivar	Gallic acid	Chlorogenic acid	Cyanidin-3-glucoside	Malvidin-3-glucoside	Ellagic acid	Syringic acid	Luteolin	Catechin
Camellia	3.05±0.08 gh	208.08±5.00 bc	221.29±8.75 hij	832.36±42.87 de	0.00±0.00 h	18.63±0.85 a	2.35±0.10 efgh	6.60±0.30 ef
Emerald	4.25±0.16 de	61.44±1.00 n	225.13±2.38 hij	753.00±27.09 def	1.53±0.01 gh	0.00±0.00 i	4.23±0.90 bcd	4.21±0.06 fg
Eureka1	2.44±0.06 i	107.82±2.00 l	297.47±6.48 efg	713.51±12.20 efg	1.50±0.06 gh	5.89±0.21 f	1.52±0.04 fgh	4.55±0.10 ef
Eureka2	6.00±0.19 b	196.34±1.00 cd	831.72±27.15 a	1483.80±22.02 a	7.46±0.22 f	9.13±0.14 cd	2.35±0.83 efgh	3.92±1.69 fgh
Farthing	4.79±0.10 cd	108.05±2.00 l	400.86±5.92 d	1095.35±46.45 c	7.33±0.12 f	13.31±0.70 b	2.23±0.10 efgh	4.43±0.18 ef
Jewel	3.73±0.10 ef	107.38±2.00 l	304.12±11.30 ef	350.63±3.70 j	22.24±1.17 bc	6.63±0.12 ef	3.05±0.08 def	16.98±0.10 b
L503	7.00±0.08 a	210.70±0.50 b	131.58±5.52 lm	531.56±15.41 hi	16.68±0.02 e	8.00±0.34 de	3.11±0.05 cdef	6.06±0.07 ef
Legacy	5.13±0.16 c	178.15±1.00 e	452.70±16.11 cd	1038.62±0.99 c	23.07±0.87 b	12.00±0.32 b	1.56±0.06 fgh	5.22±0.40 ef
Magnifica	2.72±0.10 ghi	137.62±2.00 h	231.42±2.32 hij	703.07±2.90 efg	18.57±0.14 de	0.00±0.00 i	6.88±0.27 a	16.84±0.35 b
Meadowlark	4.17±0.17 e	166.60±1.00 ef	292.20±13.79 efg	1281.83±6.07 b	1.79±0.09 gh	9.32±0.36 cd	4.82±0.06 b	7.07±0.14 e
Misty	1.53±0.08 j	161.83±1.50 fg	162.84±2.23 kl	757.44±32.27 def	29.06±1.06 a	0.00±0.00 i	2.29±0.04 efgh	20.75±0.94 a
Rocio	5.75±0.08 b	154.41±3.50 fg	223.67±1.64 hij	702.28±25.73 efg	1.49±0.03 gh	3.23±0.04 g	4.71±0.18 bc	1.13±0.05 i
Rossini	1.74±0.01 j	93.17±2.50 m	483.19±6.55 c	1001.84±5.61 c	2.18±0.06 gh	9.79±0.14 c	1.68±0.07 fgh	1.60±0.04 ghi
Scintilla	4.98±0.13 c	152.80±2.00 g	265.12±7.25 fgh	863.25±36.48 d	19.71±0.91 cd	0.79±0.03 i	2.50±0.02 efg	1.40±0.01 hi
Springhigh	4.00±0.09 e	128.97±1.00 hij	242.17±9.64 ghi	638.55±0.09 fgh	1.04±0.03 gh	7.17±0.29 ef	0.76±0.00 h	4.43±0.03 ef
Star	4.28±0.02 de	123.99±2.50 ijk	174.87±7.70 jkl	611.87±12.06 gh	0.00±0.00 h	6.34±0.20 ef	2.12±0.03 fgh	6.30±0.29 ef
Suziblue	2.45±0.05 hi	114.01±2.50 kl	105.37±4.77 m	438.40±5.54 ij	3.06±0.10 g	1.48±0.02 hi	0.85±0.04 h	10.03±0.47 d
Twilight	3.31±0.07 fg	223.89±2.00 a	658.51±3.87 b	1048.62±36.03 c	21.00±0.71 bcd	19.69±0.19 a	3.81±0.13 bcde	4.00±0.14 fgh
Unknown	2.94±0.14 ghi	192.43±3.00 d	207.41±7.58 ijk	546.85±26.62 hi	30.14±1.05 a	5.55±0.26 f	3.00±0.04 def	12.91±0.57 c
Ventura	6.74±0.00 a	136.09±1.00 hi	337.39±13.76 e	432.86±16.98 ij	7.79±0.02 f	2.73±0.03 gh	1.11±0.01 gh	4.29±0.10 fg
Windsor	3.66±0.02 ef	122.48±1.00 jk	434.04±0.93 cd	1362.92±18.88 ab	9.02±0.20 f	0.00±0.00 i	4.21±0.10 bcd	4.71±0.21 ef

Chlorogenic acid emerged as the predominant phenolic acid across all cultivars (**Table 4**), with concentrations ranging from 61.44 mg/100 g DW in ‘Emerald’ to 223 mg/100 g DW in ‘Twilight’, representing a 3.6-fold variation. The balloon plot visualization (**Figure 5**) clearly demonstrates that chlorogenic acid exhibited the highest overall concentrations and greatest variability among phenolic acids, with ‘Twilight’ (223 mg/100 g DW), ‘L503’ (210 mg/100 g DW), and ‘Camellia’ (208 mg/100 g DW) accumulated significantly higher chlorogenic acid concentrations. Chlorogenic acid was identified as the most abundant phenolic acid in lowbush blueberries, being approximately 100 times higher in concentration than either caffeic or ferulic acids (Gibson et al., 2013) Previous studies reported chlorogenic acid as the predominant phenolic acid in both lowbush (0.44 mg/g fresh weight) and highbush blueberries (0.13 mg/g fresh weight) (Wang et al., 2014), which when converted to dry weight basis aligns with our findings. The variation in chlorogenic acid content among cultivars is consistent with previous reports indicating genotype-dependent differences in phenolic acid accumulation (Herniter et al., 2023(. Gallic acid concentrations varied from 1.53 mg/100 g DW in ‘Misty’ to 7.00 mg/100 g DW in ‘L503’, while syringic acid displayed a distinctive binary distribution pattern visible in **Figure 5**, with ‘Twilight’ (19.69 mg/100 g DW) and ‘Camellia’ (18.63 mg/100 g DW) showing prominent accumulation, whereas nine cultivars showed no detectable amounts (**Table 4**). Seven phenolic acids including gallic acid, chlorogenic acid, caffeic acid, syringic acid, and ferulic acid were quantified in blueberry polyphenol fractions using HPLC-MS/MS, with chlorogenic acid being predominant (Su et al., 2017).

Ellagic acid exhibited the most dramatic variation among phenolic acids, from undetectable levels in ‘Eureka’, ‘Star’, and ‘Windsor’ to exceptionally high concentrations in ‘Unknown’ (30.14 mg/100 g DW) and ‘Misty’ (29.06 mg/100 g DW), as clearly depicted by the contrasting circle sizes in **Figure 5**. This represents over 30-fold variation, suggesting cultivar-specific regulation of ellagitannin hydrolysis pathways. Catechin content showed significant variation among cultivars (**Table 4**), with ‘Misty’ accumulating the highest concentration (20.75 mg/100 g DW), followed by ‘Jewel’ (16.98 mg/100 g DW) and ‘Magnifica’ (16.84 mg/100g DW), while ‘Rocio’ contained minimal amounts (1.13 mg/100 g DW), representing an 18-fold difference. The balloon plot (**Figure 5**) illustrates this variation with notably larger circles for ‘Misty’ and minimal representation for ‘Rocio’. Luteolin, representing the flavonoid class, ranged from 0.76 mg/100 g DW in ‘Springhigh’ to 6.88 mg/100 g DW in ‘Magnifica’ (**Table 4**). Comprehensive metabolic profiling in blueberry identified 33 health-related phytochemicals belonging to four major groups of flavonoids and phenolic acids, with substantial variation within and between ploidy levels (Mengist et al., 2020).

Previously, HPLC analysis of blueberries revealed nine phenolic acids including gallic acid, protocatechuic acid, p-hydroxybenzoic acid, vanillic acid, caffeic acid, p-coumaric acid, ferulic acid, ellagic acid, and cinnamic acid, along with various flavonoids (Huang et al., 2012). The diversity in phenolic profiles among our 21 cultivars aligns with these comprehensive characterizations. Our findings are consistent with recent studies on blueberry phenolic composition. A comprehensive HPLC-MS/MS method for 36 phenolic compounds revealed that blueberry exhibited higher contents of anthocyanins, flavonols and phenolic acids compared to other berries (Mustafa et al., 2022). Analysis of 14 highbush blueberry cultivars using UPLC-PDA-ESI-MS/MS identified 75 bioactive compounds, with anthocyanins and phenolic acids being predominant (Lachowicz-Wiśniewska et al., 2024). The substantial variation observed in individual phenolic compounds (**Table 4**, **Figure 5**)-ranging from 3.6-fold for chlorogenic acid to 30-fold for ellagic acid - underscores significant genetic diversity available for breeding programs. These findings provide critical selection criteria for breeding programs. The ‘Eureka’ × ‘Twilight’ cross could potentially combine superior anthocyanin accumulation with enhanced phenolic acid content. Similarly, ‘Misty’ represents valuable germplasm for both catechin and ellagic acid enhancement.

### Correlations among metabolites

**Figure 6:**
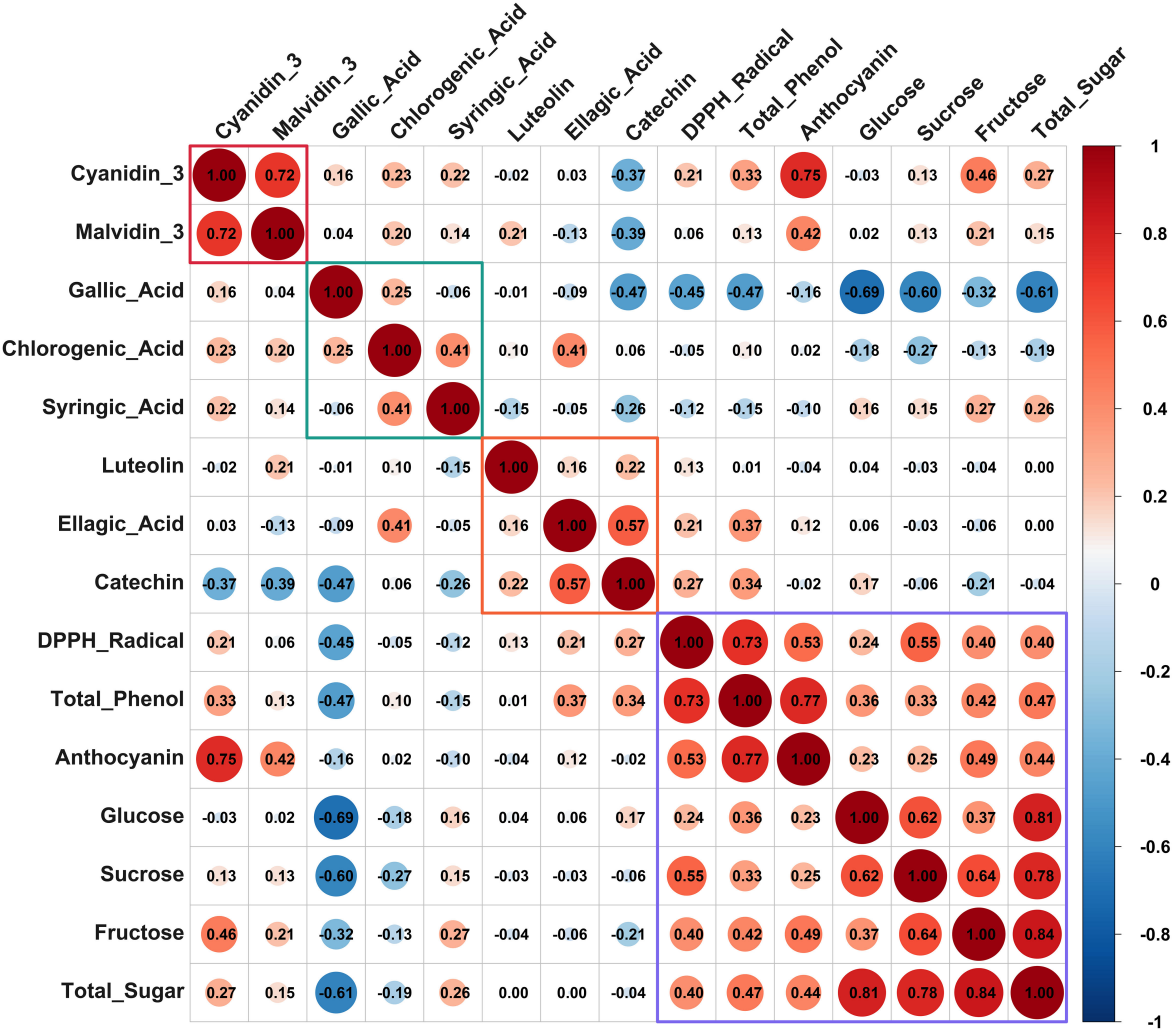
Pearson correlation matrix of phenolic compounds, sugars, and antioxidant activities in 21 blueberry cultivars.

Pearson correlation analysis revealed complex metabolic relationships among phenolic compounds, sugars, and antioxidant activities across the 21 blueberry cultivars, as illustrated in the correlation matrix (**Figure 6**). The analysis identified distinct patterns of co-accumulation, metabolic independence, and antagonistic relationships that reflect genotypic variation in secondary metabolism. Total phenolic content exhibited a strong positive correlation with DPPH radical scavenging capacity (r=0.73), validating the direct contribution of phenolic compounds to free radical scavenging mechanisms. Similarly, total anthocyanin content showed a moderate correlation with DPPH radical (r=0.53), indicating that anthocyanins constitute a significant fraction of bioactive compounds responsible for antioxidant capacity. Notably, total phenolic content and total anthocyanin demonstrated a strong positive correlation (r=0.77), reflecting the substantial contribution of anthocyanins to the overall phenolic pool in blueberry fruit.

The strong correlation between cyanidin-3-glucoside and malvidin-3-glucoside (r=0.72) suggests coordinated accumulation of these anthocyanins, possibly reflecting shared biosynthetic precursors and similar accumulation patterns among cultivars. Among phenolic compounds, catechin and ellagic acid displayed a moderate positive correlation (r=0.57), potentially reflecting similar accumulation patterns within the phenylpropanoid-flavonoid pathway. Conversely, gallic acid exhibited strong negative correlations with catechin (r=-0.47), glucose (r=-0.69), sucrose (r=-0.60), and total sugar (r=-0.61), suggesting metabolic trade-offs and competitive carbon allocation between the shikimate pathway and primary carbohydrate metabolism. Luteolin demonstrated weak associations with most metabolites, indicating genotype-specific variation in flavone accumulation independent of other phenolic pathways. Sugar metabolism showed strong internal coordination, with total sugar content highly correlated with individual sugars: fructose (r=0.84), glucose (r=0.81), and sucrose (r=0.78). Moderate positive correlations emerged between total phenolic content and individual sugars: fructose (r=0.42), glucose (r=0.36), and sucrose (r=0.33), suggesting metabolic plasticity wherein cultivars with enhanced photosynthetic capacity or carbon allocation simultaneously accumulate both primary and secondary metabolites. This phenolic-sugar coordination may reflect genotypic differences in source-sink relationships and the availability of carbon skeletons for the shikimate and phenylpropanoid pathways. These correlation patterns align with previous studies reporting coordinated anthocyanin-antioxidant relationships in blueberry cultivars (Moyer et al., 2002; Li et al., 2017). The strong phenolic-antioxidant correlations observed here support findings that anthocyanins and phenolic acids are primary contributors to antioxidant capacity in Vaccinium species. Sugar-phenolic co-accumulation has been documented during blueberry fruit ripening, where sustained carbon import supports simultaneous anthocyanin accumulation and sugar accumulation (Zifkin et al., 2012; Acharya et al., 2024). The observed negative correlations between gallic acid and sugars are consistent with metabolic channeling within the phenylpropanoid pathway, where substrate competition can create trade-offs between biosynthetic branches (Günther et al., 2020).

### Principal component analysis

**Figure 7:**
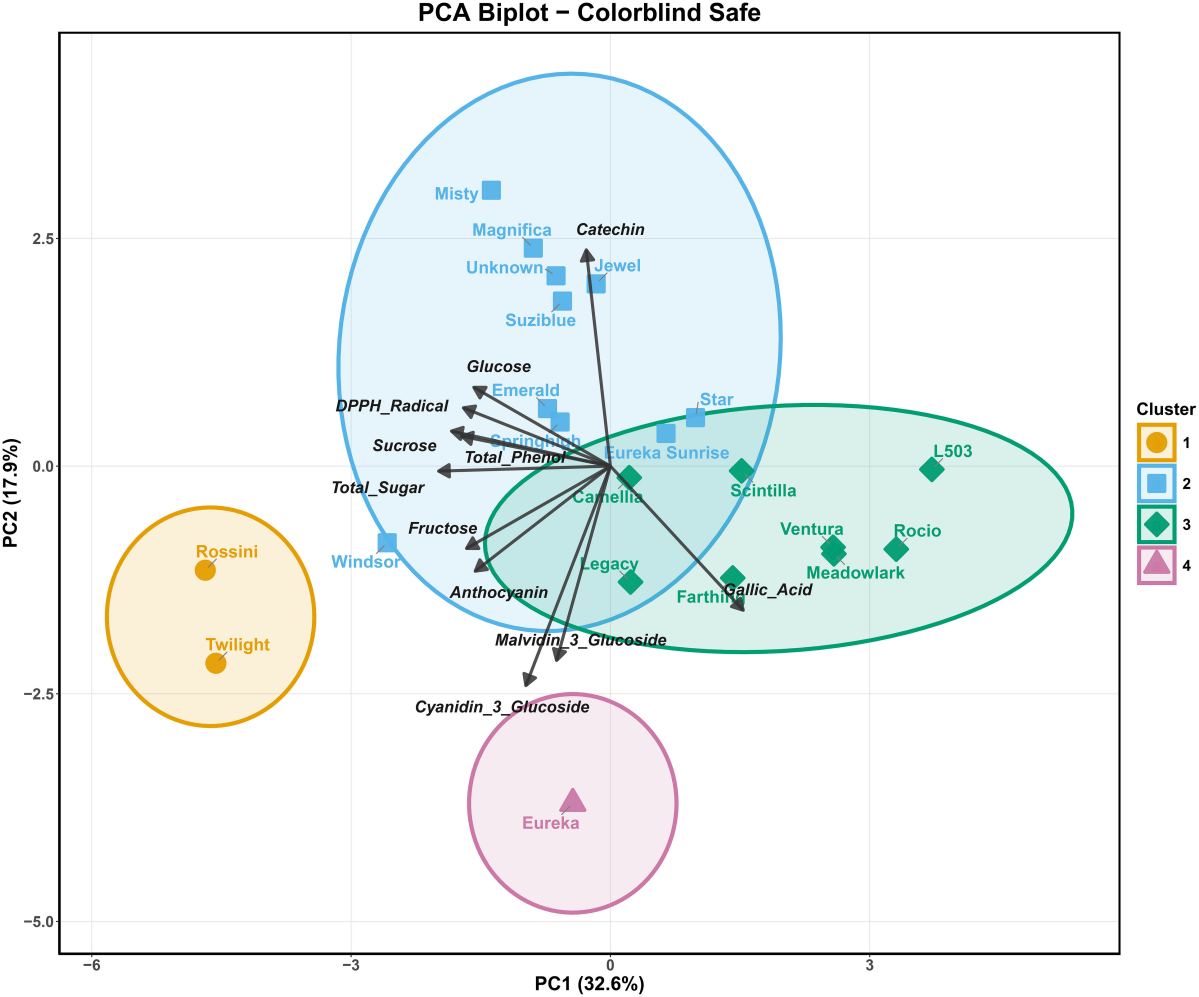
Principal component analysis (PCA) biplot of 21 blueberry cultivars based on biochemical profiles.

Principal component analysis revealed four distinct biochemical clusters among the 21 blueberry cultivars, with PC1 and PC2 explaining 32.6% and 17.5% of total variance, respectively (50.1% cumulative variance) (**Figure 7**).

Cluster 1 (orange), comprising only Rossini and Twilight, occupied the extreme negative PC1 space and was strongly associated with sugar accumulation traits (total sugars, fructose, glucose, sucrose) and anthocyanin content. The extreme positioning of these two cultivars in the negative PC1 region indicates the most pronounced metabolic prioritization toward carbohydrate storage among all genotypes examined. This sugar-dominant metabolic profile reflects enhanced sink strength and efficient carbon allocation toward hexose accumulation during fruit development.

Cluster 2 (blue), consisting of Misty, Magnifica, Unknown, Suziblue, Emerald, Springhigh, Windsor, Star, and Eureka Sunrise, was predominantly positioned in the positive PC2 space and characterized by elevated total phenolics, DPPH radical scavenging activity, and catechin content. This cluster demonstrates superior antioxidant capacity driven by coordinated accumulation of multiple phenolic sub-classes, particularly flavanols. The strong positive PC2 loadings for antioxidant parameters indicate that Misty, Magnifica, and Unknown exhibit the highest antioxidant potential among the evaluated cultivars. Within this cluster, cultivars displayed varying PC1 positions, with Star positioned in the positive PC1 space, suggesting additional metabolic complexity beyond the primary antioxidant-driven phenotype.

Cluster 3 (green), containing Jewel, L503, Scintilla, Ventura, Rocio, Meadowlark, Farthing, and Legacy, was distributed primarily in the positive PC1 and negative to neutral PC2 space. This cluster was strongly associated with gallic acid accumulation, with cultivars positioned along the positive PC1 axis showing the highest gallic acid content. The distinct separation of this cluster along PC1 reflects elevated gallic acid accumulation. L503 and Rocio, positioned at the most positive PC1 values, represent the genotypes with maximum gallic acid content.

Cluster 4 (pink), represented uniquely by Eureka, formed an isolated group in the extreme negative PC2 space with intermediate PC1 positioning. The singular positioning of Eureka suggests a distinctive metabolic profile characterized by elevated cyanidin-3-glucoside accumulation, as evidenced by its strong association with the cyanidin-3-glucoside loading vector. This unique biochemical signature indicates a distinctive anthocyanin accumulation pattern, with ‘Eureka’ showing preferential accumulation of cyanidin-3-glucoside compared to other cultivars.

The biplot reveals distinct metabolic trade-offs among cultivar groups. Sugar metabolites (total sugars, fructose, glucose, sucrose) are tightly co-aligned in the negative PC1 space, positioned opposite to gallic acid along the PC1 axis. This arrangement reflects the metabolic divergence between Cluster 1 (sugar-dominant) and Cluster 3 (gallic acid-dominant), suggesting competitive carbon allocation between primary carbohydrate metabolism and secondary metabolite accumulation. The antioxidant parameters (DPPH radical scavenging and total phenolics) cluster together in the positive PC2 space, strongly associated with catechin and driving the separation of Cluster 2. The positioning of anthocyanin compounds (cyanidin-3-glucoside, malvidin-3-glucoside) near the biplot origin suggests these metabolites contribute variability across multiple principal components rather than defining a single metabolic axis.

Similar PCA-based cultivar differentiation has been reported in comprehensive blueberry germplasm evaluations across multiple ploidy levels (Mengist et al., 2020), highbush blueberry diversity assessments (Lachowicz-Wiśniewska et al., 2024), and comparative anthocyanin profiling studies (Li et al., 2016). The metabolic divergence observed among the four clusters in the present study demonstrates substantial genotypic variation in carbon partitioning between primary and secondary metabolism. The inverse relationship between sugar accumulation (Cluster 1) and gallic acid content (Cluster 3) along PC1 indicates potential metabolic competition for carbon precursors. Furthermore, the isolation of Eureka in Cluster 4 with elevated cyanidin-3-glucoside suggests cultivar-specific differences in anthocyanin accumulation patterns, which has important implications for both fruit pigmentation intensity and bioactive compound profiles relevant to human health benefits.

### Hierarchical clustering

**Figure 8:**
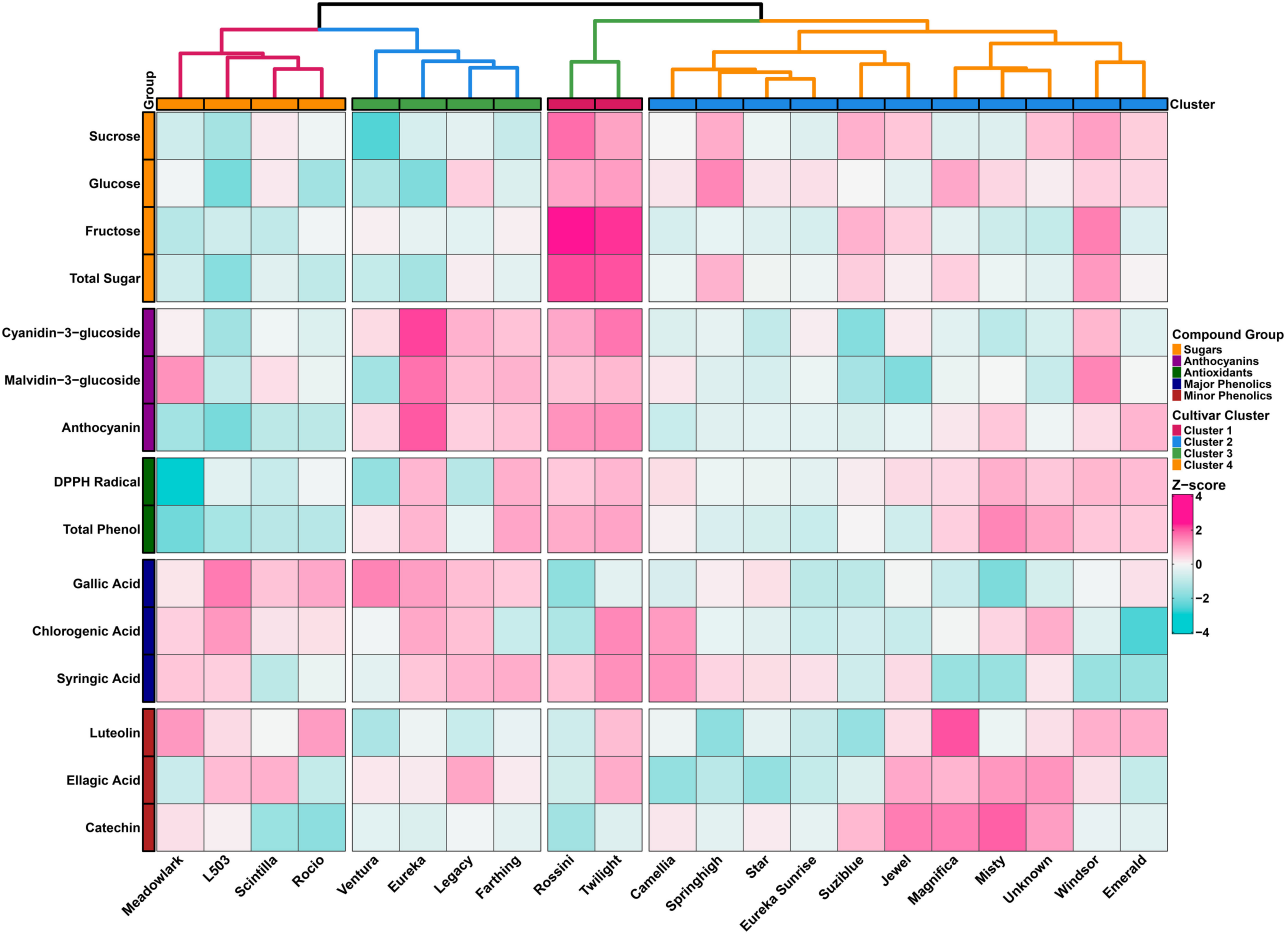
Heatmap showing hierarchical clustering of 21 blueberry cultivars based on standardized Z-scores of 15 biochemical compounds.

Heatmap analysis based on Z-score standardization revealed distinct metabolic patterns across 21 blueberry cultivars for 15 biochemical compounds, grouped into five categories: sugars, anthocyanins, antioxidants, major phenolics, and minor phenolics. Hierarchical clustering identified four major cultivar groups with characteristic biochemical signatures, demonstrating substantial genotypic variation in both primary and secondary metabolism (**Figure 8**).

Cluster 1 (Meadowlark, L503, Scintilla, Rocio) exhibited a distinctive metabolic profile characterized by low Z-scores (negative values from -2 to -4, shown in cyan) for sugar fractions, particularly sucrose and glucose. Simultaneously, this cluster displayed elevated levels of anthocyanins, especially malvidin-3-glucoside (shown in magenta with positive Z-scores). However, antioxidant activity in this cluster showed variable patterns, with some cultivars displaying moderate to low DPPH radical scavenging capacity and total phenol content. This suggests that while anthocyanin content is high, the overall antioxidant capacity may be influenced by the balance of different phenolic compounds rather than anthocyanins alone.

Cluster 2 (Ventura, Eureka, Legacy, Farthing) presented a metabolic profile with low to moderate sugar content but distinctively high antioxidant activity (DPPH radical scavenging and total phenol), shown as magenta cells with positive Z-scores. These cultivars also exhibited elevated levels of major phenolics (gallic acid, chlorogenic acid, syringic acid) and minor phenolics (catechin, ellagic acid), alongside high anthocyanin accumulation. This metabolic signature indicates coordinated accumulation of multiple phenolic compound classes.

Cluster 3 (Rossini, Twilight) presented a contrasting pattern with consistently high sugar content across all fractions (sucrose, glucose, fructose, total sugar), shown as magenta cells with Z-scores ranging from +2 to +4. These cultivars maintained moderate anthocyanin levels but exhibited variable phenolic acid profiles. This metabolic signature indicates prioritized carbon partitioning toward carbohydrate accumulation.

Cluster 4 (Camellia, Springhigh, Star, Eureka Sunrise, Suziblue, Jewel, Magnifica, Misty, Unknown, Windsor, Emerald) represented the largest group with generally moderate to elevated sugar content and distinctively high minor phenolic accumulation, particularly catechin. Conversely, this cluster exhibited significantly reduced anthocyanin content (shown in cyan with negative Z-scores) and generally lower antioxidant activity. Sugar profiles varied within this cluster, with some cultivars showing moderate to high fructose content. This pattern suggests preferential accumulation of flavanols rather than anthocyanins in these cultivars.

The relationship between sugars and secondary metabolites observed across clusters reflects carbon partitioning dynamics, where photosynthetic carbon is allocated between primary (growth-related) and secondary (defense-related) metabolism. Similar patterns of coordinated sugar and anthocyanin accumulation during fruit ripening have been reported in *Vaccinium* species (Jaakola, 2013; Zifkin et al., 2012). The antioxidant activity observed across cultivars reflects the complex interplay of various phenolic compounds, not solely anthocyanin content. Environmental factors, particularly light quality, can modulate anthocyanin profiles in *Vaccinium* species, though the genotypic variation observed in this study under uniform conditions highlights the primary role of genetic factors (Zoratti et al., 2015).

From a breeding perspective, the metabolic clustering provides clear targets for cultivar improvement. Cluster 1 cultivars with high anthocyanin content (Meadowlark, Scintilla, Rocio) serve as valuable germplasm for biofortification programs targeting enhanced pigment accumulation. Cluster 2 cultivars (Ventura, Eureka, Legacy, Farthing) offer exceptional combinations of high antioxidant activity and comprehensive phenolic profiles, ideal for functional food development. Cluster 3 cultivars (Rossini, Twilight) provide high-sugar phenotypes valuable for fresh market applications prioritizing sweetness. The large Cluster 4 group with elevated catechin content may be targeted for breeding programs focusing on flavanol-rich functional foods.

## Conclusion

This comprehensive biochemical evaluation of 21 highbush blueberry cultivars has revealed substantial genetic diversity in both primary and secondary metabolite composition, with all measured parameters exhibiting highly significant (p<0.001) cultivar-dependent variation. The observed ranges—2.7-fold in total phenolic content, 4.4-fold in anthocyanin accumulation, 2.9-fold in total sugars, and up to 30-fold in individual phenolic compounds—demonstrate the extensive metabolic plasticity available for breeding exploitation in *Vaccinium corymbosum* germplasm. The strong positive correlation between total phenolic content and antioxidant capacity (r=0.73) confirms that phenolic compounds serve as primary determinants of radical scavenging capacity in blueberry fruits. Additionally, the strong correlation between cyanidin-3-glucoside and malvidin-3-glucoside (r=0.72) indicates coordinated accumulation of major anthocyanins among cultivars. The moderate positive associations between phenolic accumulation and sugar content (r=0.33–0.42) demonstrate that selection for enhanced nutritional quality need not compromise fruit palatability, enabling integrated breeding strategies that simultaneously improve health-promoting properties and sensory attributes. Principal component analysis (50.1% cumulative variance) successfully differentiated cultivars into four metabolically distinct groups: sugar-anthocyanin co-accumulators (‘Rossini’, ‘Twilight’), superior antioxidant types (‘Misty’, ‘Magnifica’, ‘Unknown’), gallic acid specialists (‘Jewel’, ‘L503’, ‘Ventura’), and cyanidin-3-glucoside specialist (‘Eureka’). This metabolic stratification reflects fundamental differences in carbon partitioning between primary and secondary metabolism. From a breeding perspective, this study identifies specific elite germplasm for targeted applications: ‘Rossini’ and ‘Twilight’ (total sugars >17.8 g/100 g DW, anthocyanins >2100 mg/100 g) represent optimal parents for fresh market cultivars combining superior sweetness with high pigmentation; ‘Eureka’ (anthocyanins 2678 mg/100 g) serves as the anthocyanin biofortification champion for functional food development; ‘Misty’, ‘Farthing’, and ‘Windsor’ (DPPH radical scavenging >67%) provide elite alleles for antioxidant capacity enhancement. Strategic hybridizations such as ‘Eureka’ × ‘Twilight’ or ‘Misty’ × ‘Rossini’ could generate progeny combining multiple superior traits. These findings establish a comprehensive biochemical framework for precision breeding in highbush blueberry, providing essential selection criteria for breeding programs targeting enhanced nutritional value, optimized antioxidant properties, and superior consumer acceptance.

## Data Availability

The raw data supporting the conclusions of this article will be made available by the authors, without undue reservation.
